# On the Origin of Large Flexibility of P-glycoprotein in the Inward-facing State*[Fn FN2]

**DOI:** 10.1074/jbc.M113.450114

**Published:** 2013-05-08

**Authors:** Po-Chao Wen, Brandy Verhalen, Stephan Wilkens, Hassane S. Mchaourab, Emad Tajkhorshid

**Affiliations:** From the ‡ Center for Biophysics and Computational Biology, Department of Biochemistry, College of Medicine, and The Beckman Institute for Advanced Science and Technology, University of Illinois at Urbana-Champaign, Urbana, Illnois 61801,; § Department of Molecular Physiology and Biophysics, Vanderbilt University Medical Center, Nashville, Tennessee 37232, and; the ¶ Department of Biochemistry and Molecular Biology, State University of New York Upstate Medical University, Syracuse, New York 13210

**Keywords:** ABC Transporter, Electron Paramagnetic Resonance (EPR), Membrane Transport, Molecular Dynamics, Protein Dynamics, Double Electron Electron Resonance Spectroscopy (DEER), P-glycoprotein

## Abstract

P-glycoprotein (Pgp) is one of the most biomedically relevant transporters in the ATP binding cassette (ABC) superfamily due to its involvement in developing multidrug resistance in cancer cells. Employing molecular dynamics simulations and double electron-electron resonance spectroscopy, we have investigated the structural dynamics of membrane-bound Pgp in the inward-facing state and found that Pgp adopts an unexpectedly wide range of conformations, highlighted by the degree of separation between the two nucleotide-binding domains (NBDs). The distance between the two NBDs in the equilibrium simulations covers a range of at least 20 Å, including, both, more open and more closed NBD configurations than the crystal structure. The double electron-electron resonance measurements on spin-labeled Pgp mutants also show wide distributions covering both longer and shorter distances than those observed in the crystal structure. Based on structural and sequence analyses, we propose that the transmembrane domains of Pgp might be more flexible than other structurally known ABC exporters. The structural flexibility of Pgp demonstrated here is not only in close agreement with, but also helps rationalize, the reported high NBD fluctuations in several ABC exporters and possibly represents a fundamental difference in the transport mechanism between ABC exporters and ABC importers. In addition, during the simulations we have captured partial entrance of a lipid molecule from the bilayer into the lumen of Pgp, reaching the putative drug binding site. The location of the protruding lipid suggests a putative pathway for direct drug recruitment from the membrane.

## Teaser

**Background:** P-glycoprotein relies on largely unknown structural changes for its transport function.

**Results:** EPR spectroscopy and simulations capture large-amplitude structural fluctuations for inward-facing P-glycoprotein.

**Conclusion:** The characterized distinct dynamics of P-glycoprotein suggests mechanistic diversity of ATP-coupled transport in ABC transporters.

**Significance:** Characterizing structural dynamics is a key step toward understanding the mechanism of this multidrug resistance transporter.

## Introduction

One major obstacle in cancer chemotherapy is the development of multidrug resistance. Cancer cells expressing such a phenotype often present various molecular pumps on the cell surface, expelling the cytotoxic drugs out of the cell (1). Many of these molecular pumps belong to the ATP binding cassette (ABC) superfamily, a class of proteins comprising one of the largest families of primary membrane transporters (2). Among various ABC transporters contributing to multidrug resistance, P-glycoprotein (Pgp,^3^ also known as MDR1 or ABCB1) is most prominent, as it confers the strongest form of resistance against a wide spectrum of chemotherapeutic drugs in many tissue types (1).

**FIGURE 1.**
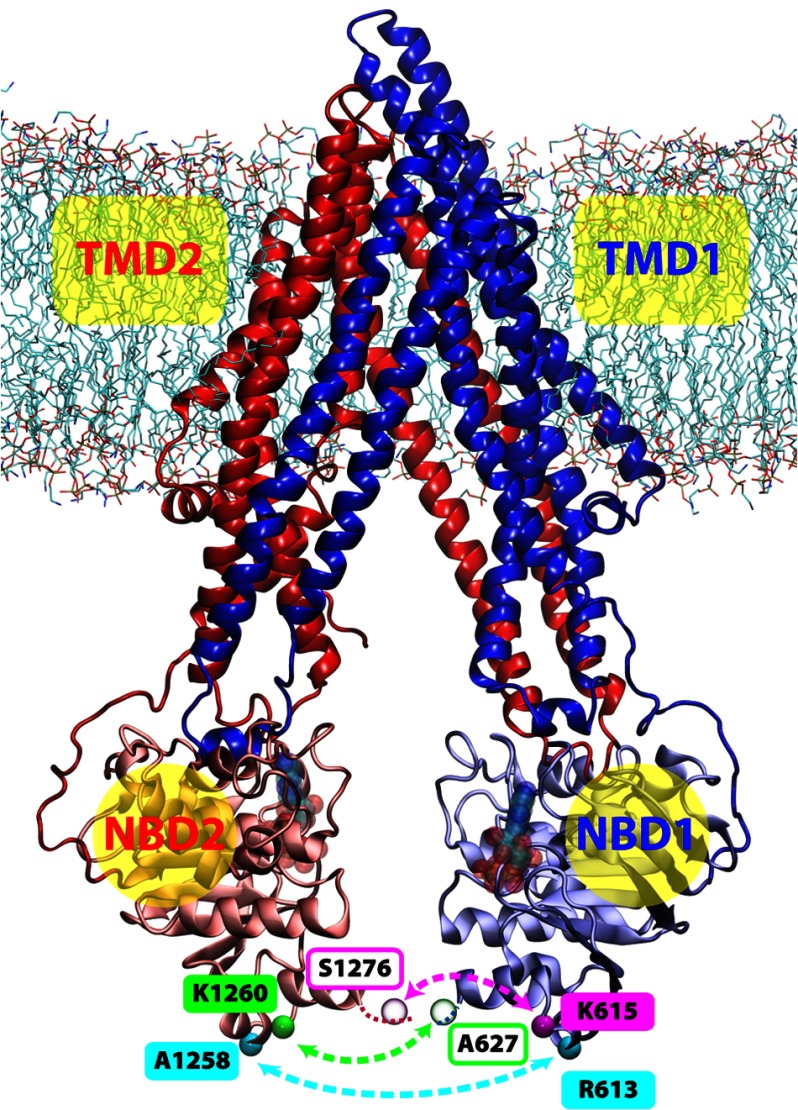
**Illustration of the membrane-bound Pgp.** The structure of Pgp is represented as ribbons in *blue* (*TMD1*), *light blue* (*NBD1*), *red* (*TMD2*), and *pink* (*NBD2*). Lipid molecules are shown as *sticks* in the following color scheme; carbon in *cyan*, oxygen in *red*, nitrogen in *blue*, and phosphorus in *brown*. The C_α_ atoms of the spin-labeled residues for DEER spectroscopy are shown and labeled in *cyan*, *green*, and *magenta spheres*, where residues not resolved in the crystal structure are in transparent spheres. The locations of the two nucleotide binding sites are shown with transparent Mg-ATP structures docked in the Mg-ATP-docked simulations.

The canonical architecture of ABC transporters is preserved in the Pgp structure (3): two “half-transporters” expressed as a single gene product, arranged in a pseudo 2-fold symmetric configuration (see Fig. 1) where each half is composed of a transmembrane domain (TMD) and a cytoplasmic nucleotide binding domain (NBD). In the crystal structure of the murine Pgp (3), the NBDs are free of any bound nucleotide, and the lumen formed within the TMDs is only accessible from the cytoplasm. Thus, the overall conformation of the transporter is in the “inward-facing” state, in contrast to the nucleotide-bound, outward-facing conformation obtained for the bacterial homolog SAV1866 (4). The putative drug binding site of Pgp is occupied by the bound inhibitor in the crystal structure (3), which is enclosed within a deep, central cleft formed between the two TMDs. The amino acid composition around the drug binding pocket explains the poly-specificity of Pgp, as the bound inhibitors are mostly coordinated non-specifically by hydrophobic residues instead of specific hydrogen bonds and/or salt bridges (3).

Structural models of Pgp have been used for drug docking to investigate its mechanism of multidrug resistance (5–18). Although the structure of Pgp has been experimentally solved only in the nucleotide-free, inward-facing state, many studies have used homology modeling to construct a model for the ATP-bound, outward-facing conformation based on the SAV1866 structure as a template (5, 6, 14, 15, 19–22). In the ATP-bound state, the two NBDs would be in close contact at the nucleotide binding sites and the bound ATP sandwiched between two highly conserved nucleotide binding motifs (termed Walker A and LSGGQ), one from each NBD monomer, similar to the conformations revealed in many dimeric structures of isolated NBDs (23–26).

Based on the structural information obtained for the nucleotide-free and the ATP-bound states, a general scheme is often used to describe the transport mechanism of ABC transporters with the canonical architecture; the two NBDs dimerize upon ATP binding and separate after ATP hydrolysis, which determines the conformations of the TMDs to alternating between the outward-facing and the inward-facing states (27, 28) due to the tight conformational coupling between the NBDs and the TMDs. The conformations revealed in the crystal structure of Pgp as well as of several bacterial homologs (including SAV1866 (4), MsbA (29), TM287/288 (30)) are all consistent with the common scheme. Although the general mechanism seems structurally clear and accurate, multiple models have been proposed to dissect the detailed transporter conformations arising during the nucleotide binding and hydrolysis steps (31, 32) as well as their connections to the substrate translocation (33–35). One of the major controversies among the different proposed mechanisms is whether the crystal structures represent physiological relevant conformational states. The presence of an apo state *in vivo* is confronted by the experimentally observed *K*_m_^ATP^ and *K*_d_^ATP^ being lower than the cellular ATP concentration (36–38), and the likelihood of a constantly present nucleotide implies that the two NBDs of Pgp may not separate to the degree shown in the crystal structure, as suggested by some FRET and cross-link studies (39, 40).

Besides the challenges to the general mechanism, whether the transport mechanism of Pgp is similar to that of other ABC transporters or even to any other ABC exporter is not well understood. For example, unlike most of its bacterial homologs that appear as a homodimer of two identical halves, Pgp is expressed as one gene product containing both halves, which makes its structure intrinsically asymmetric. Yet, when compared with asymmetric ABC exporters, Pgp has two fully functional NBDs, in contrast to many multidrug-resistant proteins in the ABCC subfamily (41), whose structural asymmetry is highlighted by the common presence of one degenerate nucleotide binding site that contains a dysfunctional catalytic dyad (42). In addition, Pgp hydrolyzes ATP at a significantly high rate in the absence of any substrate, and the drug substrates only stimulate the hydrolysis rate at 5–10-fold from the basal ATPase activity (43), in stark contrast to some other ABC transporters where transport substrates could accelerate the ATPase activity by several orders of magnitude, *e.g.* the maltose transporter (44).

Molecular dynamics (MD) simulations have been extensively used to study the dynamics of full ABC transporters in the membrane (45–58). Here, we use a combination of MD simulations and double electron-electron resonance (DEER) EPR spectroscopy to investigate the dynamics of Pgp in a membrane environment. We found that the transporter exhibits a large degree of conformational fluctuation in the inward-facing state regardless of the presence or absence of the nucleotide. Both the MD simulations and the DEER measurements indicate that the distance between the two NBDs spans a range covering at least 20 Å in the apo state. The high degree of structural fluctuation of Pgp appears to originate from the helical kinking and/or unwinding within the TMDs but is amplified outside the membrane, thus appearing most pronounced in the NBDs. The flexible nature of the Pgp structure may be directly related to its prominent basal ATPase activity; Pgp is able to form a nucleotide-bound NBD dimer even in the absence of the transport substrate because of the structure flexibility that can partially decouple the NBDs from the TMDs during the conformational transitions.

## EXPERIMENTAL PROCEDURES

### 

#### 

##### Protein Purification and Labeling

Three pairs of cysteine residues were introduced into the mouse cysteine-less Pgp (59) at the C-terminal region of each NBD. Based on the sequence alignment of the two NBDs, one of the three cysteine pairs (Cys-613—Cys-1258) was placed in a symmetric design, whereas the other two pairs (Cys-615–Cys-1276 and Cys-627–Cys-1260) were in asymmetric arrangements. The two asymmetric pairs were initially designed to address conformational flexibility of the NBDs by disulfide cross-linking, and here they are able to provide more information about the relative positions of the NBDs and their conformational flexibility. The expression and purification of double-cysteine mutants were as described elsewhere (60, 61). After elution from nickel-affinity resin, protein was labeled with three sequential additions of 30-fold excess of methanethiosulfonate spin label on ice. Pgp was further purified and separated from the unbound spin label compound by size exclusion chromatography with a Superdex 200 column (10 × 300 mm) in 50 mm Tris-HCl with pH 7.4, 50 mm NaCl, 20% glycerol, and 0.05% *n*-dodecyl-β-d-maltopyranoside. Protein was concentrated by ultrafiltration using a membrane with a 100 kDa molecular mass cutoff, and the protein concentration was determined by UV/visible absorption at 280 nm with an extinction coefficient of 115,600 m^−1^·cm^−1^. To reconstitute Pgp in mixed micelles, Pgp was mixed with lipids and detergent at a molar ratio of 1:105:12.7 of protein:lipid:detergent, where the lipids were a mixture of *Escherichia coli* polar lipids and egg phosphatidylcholine in a 3:2 ratio, and the detergent was *n*-dodecyl-β-d-maltopyranoside. The final glycerol concentration was 23.4% by volume for cryoprotection. Pgp was incubated for 5 min at 37 °C to mimic the physiological active temperature of the protein, which has been shown necessary for maximal ATPase turnover and ATP occlusion (62).

##### EPR Spectroscopy

All measurements were carried out on a Bruker ELEXSYS 580 FT-EPR spectrometer equipped with a Super Q-FT bridge operating at Q-band microwave frequency (34 GHz). The DEER experiments used the 4-pulse DEER sequence at a temperature of 83 K. The π/2 and π detection pulses had widths of 12 and 32 ns, respectively. The pump pulse was set to the low field maximum, and the observed pulse was typically 60 MHz below the pump frequency corresponding to the central resonance line. Data collection time varied with the echo evolution time but was typically 12 h. DEER signals were analyzed by the Tikhonov regularization with the software DeerAnalysis 2011 to determine distance distributions *P*(*r*) (63).

##### Modeling and Simulations

A more detailed description of the procedures used for the construction of membrane-embedded Pgp models, docking of Mg-ATP into Pgp for MD simulations, the simulations of membrane-bound Pgp, and the analyses of the simulation results, is provided in supplemental materials. In short, four different membrane-embedded Pgp systems based on the crystal structure of the inward-facing, apoPgp (PDB code 3G5U; Ref. 3) were constructed with different initial placements of the protein in the lipid bilayer (Fig. 1, termed Systems 1–4 hereafter). After initial equilibration of the protein/membrane systems (typically on the order of 5 ns), each system was simulated for 50 ns under two different conditions: apo and with docked Mg-ATP.

Mg-ATP was docked at the two Walker A motifs of the NBDs. To establish a stable nucleotide-bound configuration of the NBDs that is maintained during the production simulations without any constraint, the Walker A motifs of Pgp were gradually modified to adopt a nucleotide-bound conformation over a course of 1.5 ns biased MD simulation in the presence of the docked Mg-ATP. During this phase, distance restraints were applied between the atoms of Mg-ATP and specific binding residues (supplemental Table S1 and Fig. S1*a*) to mimic the Mg-ATP-bound state of a bacterial ABC exporter (HlyB) that has been crystallized as an isolated NBD dimer (PDB code 1XEF; Ref. 25). The restraints were gradually removed during the 1.5-ns time span, and the following production simulations were carried out in equilibrium with the docked Mg-ATP until *t* = 50 ns. The docked nucleotides remain within the nucleotide binding sites after the removal of the restraints, indicating that the Walker A motifs have adopted a stable nucleotide binding conformation during the docking procedure. The root mean square deviation of 9 Walker A C_α_ atoms in Pgp (Gly-423—Thr-431 and Gly-1066—Thr-1074) with respect to the corresponding atoms in HlyB-NBD (Gly-502—Thr-510, the template used for nucleotide docking), is mostly 1 Å or lower in the nucleotide-docked simulations, in contrast to their higher root mean square deviation of ∼2 Å under the nucleotide-free condition (supplemental Fig. S1*b*).

The conformation and structural flexibility of Pgp captured in the simulations were evaluated and analyzed by monitoring (*a*) the center-of-mass (CoM) distance between the two NBDs, (*b*) the CoM distance between two highly conserved ATP binding motifs that would complete a nucleotide binding site upon NBDs dimerization (note that Pgp has two distinct nucleotide binding sites), (*c*) the root mean square fluctuation (r.m.s.f.) of the C_α_ atoms, and (*d*) the hydrogen bond frequency between residues *i* and *i*+4 in the transmembrane helices. Sequence alignment of ABC exporters is obtained by joining a set of local alignments where each one only aligns one or two transmembrane helices of the transporters. The Gly/Pro contents in TMDs of ABC exporters were calculated based on 33,741 aligned sequences of TMDs of ABC exporters, which were retrieved from entry PF00664 of the database Pfam (64).

## RESULTS AND DISCUSSION

### 

#### 

##### Dynamics of P-glycoprotein in Membrane

In this study the dynamics of the inward-facing Pgp in a membrane environment was investigated using a combination of experimental and simulation approaches. Here we use DEER spectroscopy to measure the distance between spin labels located in each NBD of Pgp and MD simulations of a membrane-embedded Pgp model to investigate the nature of the elements responsible for the dynamics of the protein and to characterize in detail Pgp conformations that gave rise to the phenomenon observed during the DEER experiments. Interestingly, a high variety of different conformations has been captured with both approaches, strongly suggesting that the structure of Pgp is highly flexible. The large conformational changes seen here are consistent with structural changes in the nucleotide-free and AMP-PNP bound structures previously depicted by other methods (65).

**FIGURE 2.**
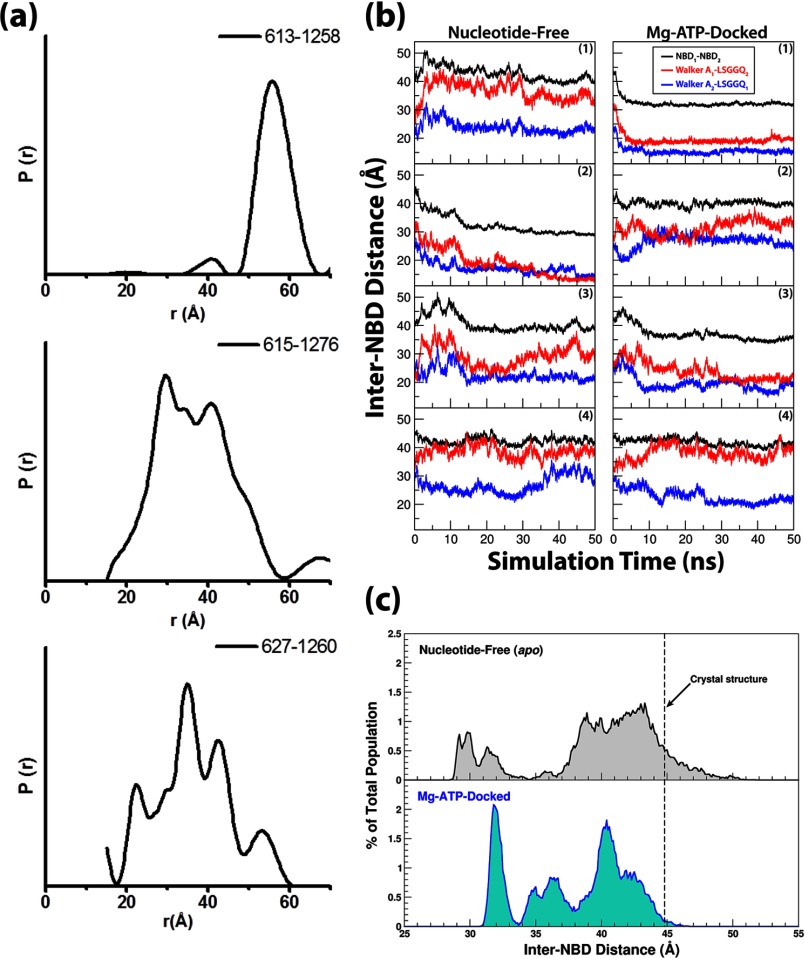
**Inter-NBD distance captured in experiments and in simulations.** *a*, distance distributions were measured by DEER spectroscopy for Cys pairs 613–1258 (*top*), 615–1276 (*middle*), and 627–1260 (*bottom*). *b*, NBD dynamics are represented by time traces of their CoM distances as well as the CoM distances between the opposing ATP-binding motifs (Walker A and LSGGQ) from different NBDs. *c*, distributions of the inter-NBD distances over all simulation trajectories under nucleotide-free and Mg-ATP-docked conditions are shown. The distributions are calculated every 0.1 Å. The distance distributions are only marginally affected after the exclusion of the first 10 ns of the trajectories from the calculations (see supplemental Fig. S7*a*), with slight changes on the average distances (38.8 Å and 37.4 Å under apo- and Mg-ATP-docked conditions, respectively).

To experimentally assess the distance distributions between the two NBDs of Pgp using DEER spectroscopy, three previously characterized double-Cys pairs (R613C/A1258C, A627C/K1260C, K615C/S1276C) (60, 61) were chosen for site-directed spin labeling. To characterize the conformations of Pgp in the inward-facing state, distances were measured in a nucleotide-free and substrate-free environment. The resulting distance distributions for all three double-Cys pairs were broad, indicating a heterogeneous conformational ensemble (Fig. 2*a* and supplemental Fig. S2). The width of the distributions, up to 40 Å, suggests large amplitude motions between the NBD sampling conformations that are both more open and more closed than the crystal structure (3). The large amplitude fluctuations of Cys-615–Cys-1276 and Cys-627–Cys-1260 were previously indirectly detected by spontaneous disulfide cross-linking that did not compromise the drug-stimulated activity of Pgp (60). The distance distribution of Cys-613—Cys-1258 has a ∼20 Å width centered at ∼58 Å. Accounting for the length of the spin label, this average distance is close to the C_β_–C_β_ separation in the crystal structure (48 Å) (3).

To simulate Pgp structure in a membrane environment, four membrane-embedded Pgp models (Systems 1–4, Fig. 1) were constructed and simulated under either nucleotide-free or Mg-ATP-docked conditions (referred to as the “apo” and the “nucleotide-docked” conditions hereafter). Each of the 8 (4 systems × 2 conditions) simulations was carried out for 50 ns. The CoM distance between the two NBDs and between the two sets of strictly conserved nucleotide binding motifs (the Walker A motif and the LSGGQ from the opposing NBD) is plotted in Fig. 2*b*. Overall, the inter-NBD distance spans a range from 28.5 to 51.7 Å, *i.e.* ∼7 Å greater and ∼16 Å smaller than the distance observed crystallographically (3). To further analyze the effect of ATP on the conformational state of Pgp, the CoM distances between the two NBDs recorded under both conditions are grouped into 0.1 Å bins and presented as population distributions (Fig. 2*c*). Although both distributions are likely incomplete due to limited simulation time, the population under the apo condition seems more broad. The population average of the NBD-NBD distance under the apo conditions is ∼2 Å larger than that of the nucleotide-docked simulations (39.6 *versus* 37.9 Å), although the difference is marginal compared with the distance change that would happen upon ATP-induced NBD dimerization. From these results, one might infer that the presence of Mg-ATP in the nucleotide binding sites facilitates NBD dimerization only by shifting the equilibrium among multiple possible conformations of the transporter rather than driving the entire process. That is, although the transporter itself is able to adapt various conformations, the presence of Mg-ATP results in a higher probability of a shorter NBD-NBD distance, thereby allowing the two NBDs to contact each other more frequently to establish specific interactions necessary for their dimerization, such as the hydrogen bond network between the LSGGQ motif and the ATP γ-phosphate and around the hydrolytic water involving residues in the Q-loop, Walker B, D-loop, and H-loop motifs (66, 67).

The distributions obtained through the experiments and those from the simulations all appear broad, suggesting a highly mobile nature of apo Pgp. In the simulations, Pgp conformations with more closed NBDs than in the crystal structure are captured more frequently, whereas a more significant population in the DEER experiment shows NBD separations greater than the crystal structure. The qualitative comparison between the results of DEER experiments and the simulations is easier with the Cys-613—Cys-1258 labeling pair. Although the experimental findings for Cys-615–Cys-1276 and Cys-627–Cys-1260 pairs are also consistent with the flexibility observed in the simulations (supplemental Fig. S3), we note that the absolute distances are not directly comparable, primarily because the spin label sites were not resolved in the structure, further supporting the inherent flexibility of this region.

The smallest NBD-NBD distances captured in the simulations under either conditions are similar, 28.5 Å for apoPgp and 30.8 Å for the Mg-ATP-docked form, and are only 2–5 Å larger than the NBD-NBD distance of a completely closed NBD dimer; a distance of 26.0–26.5 Å is captured in the crystal structures of several other ABC exporters, *e.g.* HlyB-NBD (25), SAV1866 (4), and MsbA (29). At such a short inter-NBD distance, the NBDs resemble a “semi-open” conformation that is annotated by the isolated NBD crystal structure of the *E. coli* maltose transporter (24). Interestingly, it seems that the closure of the NBDs can be achieved without the presence of nucleotide in Pgp. In addition to the partial NBD closure shown in the simulations, the DEER-measured distances also indicate that at least a fraction of the NBDs in the experiments has been captured in dimer-like conformations even in the absence of the nucleotide. A minor population in the Cys-613—Cys-1258 experiment shows an inter-NBD distance of ∼40 Å, which matches the distance between the corresponding residues in the outward-facing structures of MsbA (29) and SAV1866 (4) (∼38 Å in both cases). A similar nucleotide-independent behavior is also exhibited in DEER experiments of MsbA (68); that is, a small portion of apoMsbA adopts distances similar to MsbA trapped in the post-hydrolysis state using ADP-vanadate. This is also in line with the fact that MsbA can be crystallized in two drastically different apo forms (29), one showing extremely large NBD separation, whereas the NBDs of the other structure are in contact with each other.

##### The Origin of Structural Flexibility of P-glycoprotein

**FIGURE 3.**
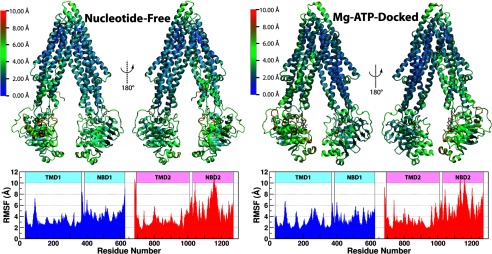
**The r.m.s.f. of C_α_ atoms of Pgp under each of the two simulating conditions.** r.m.s.f. was calculated for all four systems combined and shown as a heat map over a representative structure. The r.m.s.f. results are not significantly altered after excluding the first 10 ns of trajectories of each system (see supplemental Fig. S7*b*).

To illustrate the structural flexibility of Pgp, the r.m.s.f. of the C_α_ atoms averaged over the four simulations under the same conditions are shown in Fig. 3. Comparing the r.m.s.f. among different parts of the transporter, it becomes evident that the membrane-spanning section of the helical bundle in the TMDs (see Fig. 1 for the membrane position) generally exhibits much lower fluctuations than the solvent-exposed parts, including those on either the cytoplasmic or the extracellular sides. The theoretical temperature factors based on the fluctuations observed in the simulations are calculated using the relationship θ = (8π^2^/3) × (r.m.s.f.)^2^ and compared with the crystallographic B factors (supplemental Fig. S4). Both sets of data indicate high flexibility of the protein. Qualitatively, the r.m.s.f. in our simulations are proportional to the temperature factors obtained in the crystal structure except for some transmembrane helices, likely due to the presence of the explicit membrane in the simulations.

The greater NBD fluctuation relative to that of the TMDs is consistent with the measured conformations of some homologs of Pgp. In the case of MsbA, the dynamic range of spin-spin distance was found to be much greater when labeling at the NBDs than in the TMDs (68, 69), and a solid-state NMR study of a bacterial multidrug exporter LmrA captured very fast tumbling that is almost exclusively attributed to the NBD motions, whereas the TMDs appear immobile under nucleotide-free conditions (70). Note that the recorded frequency of the “fast” LmrA-NBD fluctuations (70) is still much slower than the motions captured in our simulations. It can, therefore, be speculated that the Pgp structure can be even more flexible than its bacterial homologs. This speculation is supported by further structural and sequence analyses of ABC exporters (details below).

**FIGURE 4.**
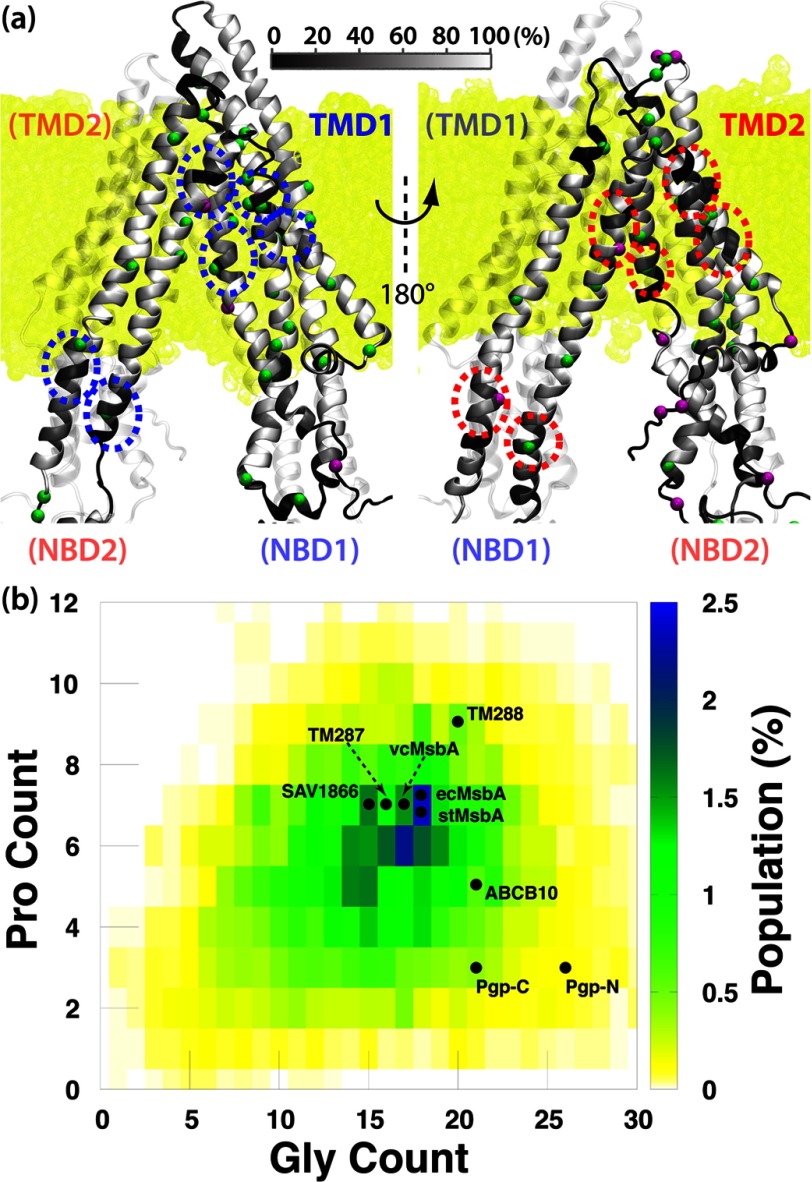
**Helical unwinding in the TMDs of Pgp.** *a*, front (*left*) and back (*right*) views of Pgp show the regions in the TMDs where the transmembrane helices unwind during the simulations. The α helices in the TMDs of Pgp are colored based on the frequency of each residue making hydrogen bonds with residue *i*+4. The C_α_ atoms of glycine (*green*) and proline (*purple*) residues are shown in *spheres*. The membrane is colored *yellow* in the background. Regions where both TMDs exhibit similar helical defect are *highlighted in circles. b*, shown is the Gly/Pro content in the TMDs of ABC exporters. The Gly/Pro counts for structurally resolved ABC exporters in the global alignment are labeled. TMD1 and TMD2 of Pgp are labeled as Pgp-N and Pgp-C, respectively. MsbA from different species (*E. coli*, *Salmonella typhimurium*, and *Vibrio cholerae*) are labeled with different prefixes.

Although the NBDs appear to exhibit greater dynamics than the TMDs, individually each NBD behaves like a rigid body with internal root mean square deviation even lower than that of the two TMDs (supplemental Fig. S5). The apparent higher intradomain root mean square deviation in the TMDs seems to be due to their hinging motions, which gets amplified and results in the greater interdomain fluctuations at the NBDs. Fig. 4*a* illustrates the positions of helical defects within the α-helical regions (calculated using STRIDE; Ref. 71) of the TMDs, where a helical defect is defined when the hydrogen bond between residues *i* and *i*+4 fails to persist for >30% of the total simulation time. Disrupted hydrogen bonds within the transmembrane helices can indicate a hinge region that might kink or unwind to produce large conformational changes, with residues farther away from the hinge naturally exhibiting greater displacements than those closer to the hinge.

Surprisingly, despite significantly different sequences between the two TMDs of Pgp, the hinge regions in the transmembrane helices of the two half-transporters are located at comparable heights with respect to the membrane (Fig. 4*a*). The transmembrane helices TM1, TM2, and TM6 in TMD1 all kink/unwind at points corresponding approximately to the midpoint of the membrane, and so do their counterparts in TMD2, namely, helices TM7, TM8, and TM12; helix TM5 in TMD1 is kinked near the cytoplasmic surface of the membrane and so is helix TM11 in TMD2; finally, in TMD1 the extracellular ends of helices TM5 and TM6 are partially unfolded, and a similar unfolding is also present for to the counterpart region in TMD2, namely, extracellular ends of helices TM11 and TM12. All these suggest that the symmetry between the two TMDs is mostly at the topological level. This topological similarity between the two TMDs, which is not apparent from their low degree of sequence homology, accounts for the comparable dynamics of these domains observed in our simulations.

Examining the primary sequences around these hinge regions, we find frequent occurrences of glycine or proline residues (labeled in Fig. 4*a*). Given the high helix-breaking propensity of these residues, their presence in the hinge regions of the TMDs might represent an important structural feature in Pgp. Interestingly, a G185V mutation in human Pgp (equivalent to Gly-181 of mouse Pgp, a glycine that bends helix TM3, supplemental Fig. S6) is well studied and shown to improve the coupling between ATP hydrolysis and transport (72). In addition, the G346C mutation in human Pgp (equivalent to Gly-342 of mouse Pgp, which causes a kink in helix TM6, supplemental Fig. S6) is also suggested to be involved in the NBD-TMD communication despite its remote location from the NBD-TMD interface (73). As both mutants exhibit reduced basal ATPase activity, it is likely that they result in increased helicity of TM3 and TM6 and thereby a more rigid TMD structure that accounts for the reduced basal ATPase activity compared with WT-Pgp. The suggested hinge role played by Gly-346 of human Pgp can also explain why the ATPase activity can only be significantly reduced by a mutation at this particular position but not elsewhere along the helix TM6 (73) even when other glycines in TM6 were targeted (G341C and G360C) (73).

A glycine residue can serve as a hinge in a transmembrane helix, providing a mechanism to switch between straight and bent helical conformations. A glycine hinge has been characterized as a conserved element of the gating mechanism in K^+^ and Na^+^ channels (74, 75), mediating the opening and closure of the channel by facilitating the bending and straightening of transmembrane helices in response to the gating signal. In particular, a study on the *Shaker* K^+^ channel has shown that a conserved glycine is required for the gating and that any residue substitution except a proline prevents the channel from opening, although the proline mutation stabilizes the open state (74), with both phenotypes likely due to restricted conformational flexibility of the transmembrane helices. In a transporter, multiple conformational states are required to allow substrate translocation. A combination of several sets of glycine residues can provide an efficient mechanism for the structure to switch between the inward-facing, outward-facing, and other intermediate states. In the case of Pgp, the glycine/proline residues may even provide extra flexibility needed to accommodate its substrates that are highly diverse in shape and size.

Interestingly, these pivotal glycines and prolines in the TMDs of Pgp do not always appear at the same positions in the aligned sequences (supplemental Fig. S6). The consistent presence of these residues in a certain region, without strict conservation of the exact position, also suggests that they play a role in protein dynamics by conferring structural flexibility rather than allowing for close helix packing. Such a role for glycine residues has been demonstrated in a mutagenesis study of the *Shaker* channel, where a mutation at a hinge glycine could be functionally compensated by a glycine mutation in the following residue (76). The unaligned glycines and prolines in Pgp are consistent with the finding that the regions of helical defects in the two TMDs are similar but not identical (Fig. 4*a*).

Expanding the structure-based sequence alignment to other TMDs of structurally known ABC exporters, we find that some of the helix-breaking glycine/proline residues are not found in other ABC exporters (supplemental Fig. S6). A systematic examination of Gly/Pro contents in the TMDs of ABC exporters also reveals significantly more Gly residues in the TMDs of Pgp than in other ABC exporters (Fig. 4*b*). Even though the Pro content in Pgp is lower, considering glycines can provide higher degree of freedoms for the backbone dihedral angles than prolines and pre-prolines (77, 78), the higher Gly content in Pgp may facilitate easier transition between the straight and bent/unwound transmembrane helices, thereby allowing Pgp to exhibit greater flexibility than its homologs. Pgp fluctuations captured in our simulations and those reported by Gyimesi *et al.* (55) are indeed larger than those reported in most simulations of a bacterial homolog SAV1866 (50–52, 56). Although this distinction might be simply due to interspecies differences of the transporter, it is important to note that SAV1866 was simulated in its outward-facing state, which can potentially have very different dynamics from the inward-facing state, the latter being the state for which Pgp simulations have been performed in the present study. Indeed, the greater flexibility of the inward-facing state of ABC exporters has been demonstrated by MD simulations of MsbA and in the same study with hydrogen-deuterium exchange experiments of BmrA (57).

In an earlier study, we simulated the *E. coli* maltose transporter and identified the coupling mechanism between its NBDs and TMDs (53). Unlike the high flexibility exhibited by Pgp, the conformational change in the TMDs of the maltose transporter in different states can be effectively described as a relative rotation between two rigid bodies, each composed of the core of one of its TMDs (79). Structural analysis of transporters in the same subfamily (type I ABC importers) further extends the definition of rigid body to the helical subdomain of the NBDs, including several conserved motifs at the NBD-TMD interface as their coupling mechanisms (53). On the other hand, structural comparison of type II ABC importers shows that only a part of their TMDs changes conformation among different states (80, 81). Comparing the TMD dynamics of ABC transporters in different subfamilies, we might speculate that they employ different mechanisms to utilize the work generated by the NBDs (through ATP binding and hydrolysis) during the substrate translocation processes.

##### Lipid Protrusion into the TMDs

**FIGURE 5.**
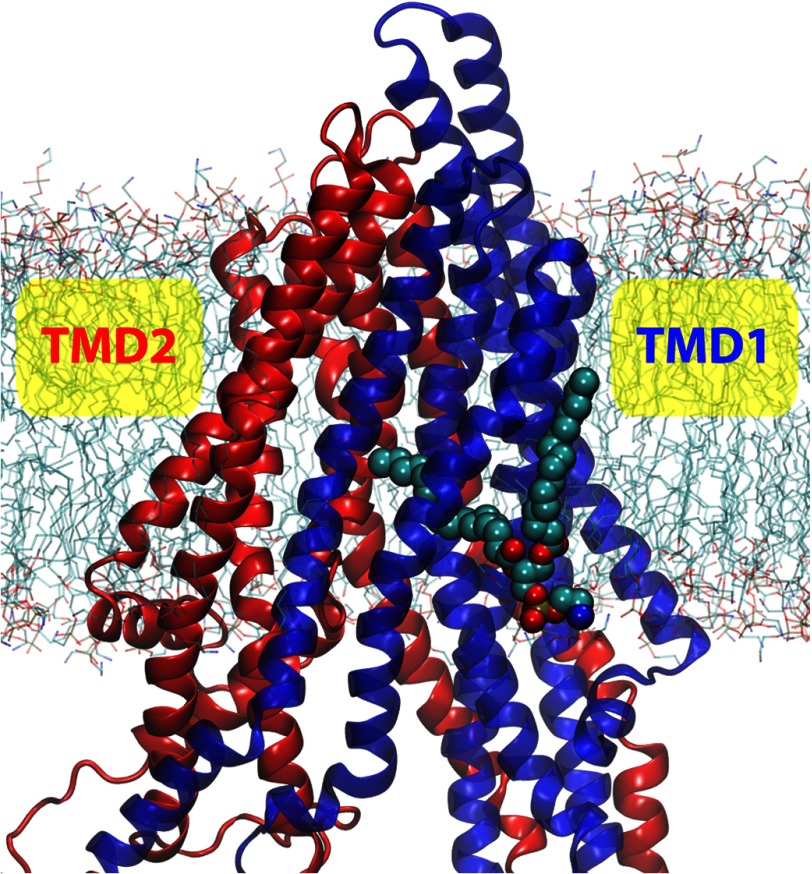
**A snapshot (*t* = 50 ns in System 1 of apo-simulations) of a lipid penetration event into the TMDs of Pgp.**

During our simulations, we captured a lipid protruding event through a putative drug portal lined with helices TM3, TM4, and TM6. Under both apo- and nucleotide-docked conditions, the oleate tail of a lipid molecule of System 1 was found to penetrate into the cleft between TM4 and TM6 and stayed there throughout the 50-ns simulation time span (Fig. 5). At least 10 carbon atoms from the end of the oleate tail entered the lumen through the portal, with the tip of the acyl chain reaching the experimentally characterized drug binding site (3). The end of the protruding lipid tail makes contacts with several experimentally confirmed drug-interacting residues, Leu-300, Ala-302, Tyr-303, and Ala-338.

P-glycoprotein has been reported to exhibit lipid flippase activity in cells (82–84) or when reconstituted into proteoliposomes (85, 86). It is suggested that the drug transport and lipid flipping activities of Pgp share the same pathway (85) as the flippase activity can be reduced by the presence of various Pgp drug substrates (82–86). The lipid protruding event captured in the simulations may present an initial step of substrate binding in Pgp. The region where the lipid tail penetrated the TMDs can provide a possible passage for drugs to enter the transporter. Interestingly, the observed penetration appears to be facilitated by the presence of several smaller residues lining this putative portal, *e.g.* Ala-225, Gly-226, Ala-229, Ala-338, Val-341, Gly-342, and Ser-345.

##### Concluding Remarks

We have characterized the highly dynamic nature of membrane-bound Pgp in the inward-facing state using both DEER spectroscopy and MD simulations. A wide range of NBD separations has been captured with both techniques, including distances longer and shorter than in the crystal structure. Although the NBDs exhibited greater deviations, the structural flexibility appears to originate from helical bending/unwinding within the TMDs. The presence of Gly/Pro residues accounts for the TMD malleability as their locations coincide the bending/unwinding regions, and several of these residues are known to suppress the basal ATPase activity once mutated. Compared with other ABC exporters, Pgp has a higher Gly/Pro content in its TMDs, suggesting greater structural flexibility. The observed flexibility of Pgp is consistent with the dynamics of several other ABC exporters and might be indicative of a primary difference in the transport mechanism from ABC importers; in contrast to a highly coordinated, substrate-dependent rigid body motions among different domains in ABC importers, the structural flexibility of Pgp has access to multiple conformational states regardless of the presence or absence of substrate and/or nucleotides, which may account for the transport independent ATPase activities. In addition, we have captured a lipid protrusion event into the TMDs, offering first evidence for a putative pathway for drug entrance into the transporter.

## Supplementary Material

Supplemental Data

## Footnotes

3 The abbreviations used are:

Pgp: P-glycoprotein
TMD: transmembrane domain
NBD: nucleotide binding domain
ABC: ATP binding cassette
CoM: center of mass
DEER: double electron-electron resonance
r.m.s.f.: root-mean-square fluctuation
AMP-PNP: adenosine 5′-(β,γ-imino)triphosphate).
