# Supplementary material for: On the Origin of Large Flexibility of P-glycoprotein in the Inward-facing State
Source: J Biol Chem. 2013 May 8;288(26):19211–20. doi: 10.1074/jbc.M113.450114 (PMC3696692; doi:10.1074/jbc.M113.450114)
Supplement: Supplemental Data [file supp_288_26_19211__index.html]

On the Origin of Large Flexibility of P-glycoprotein in the Inward-facing State — Conformational Dynamics of P-glycoprotein — Supplemental Data 

# On the Origin of Large Flexibility of P-glycoprotein in the Inward-facing State

## Supplemental Data

**Files in this Data Supplement:**

- Supplemental Material (.pdf, 4.6 MB) - Supplemental file includes expanded methods and supplementary figures.
