# Supplementary material for: On the Origin of Large Flexibility of P-glycoprotein in the Inward-facing State
Source: J Biol Chem. 2013 May 8;288(26):19211–20. doi: 10.1074/jbc.M113.450114 (PMC3696692; doi:10.1074/jbc.M113.450114)
Supplement: Supplemental Data [file supp_M113.450114_supplement-re-referenced.pdf]

Supplemental Materials

Construction of Simulation Systems

The unliganded crystal structure of Pgp (PDB:3G5U [1]) is used for the construction of membrane-embedded Pgp models that were used in the MD simulations. The models were based on the polypeptide chain A of the PDB file, including the two half-transporters (V33–T626, L684–A1271), with the C952A mutation reverted; the protein-associated mercury ions were removed since they were introduced only for phasing purpose. The unresolved N- and C-terminal regions (M1–A32, G1272–H1284) were not modeled since they only contain the signal anchor and the His-tag sequences. The linker peptide between the two half-transporters (A627–A683) was not modeled either, since several previous studies have shown that the function of Pgp does not rely on the linkage between the two halves [2–5]. Since the Pgp models do not include a complete polypeptide chain, the N- and C-termini of both the two half-transporters in the models were capped individually with acetamide (V33 and L684) and N-methylamide (T626 and A1271) groups, in order to avoid introducing artificial charges at these positions.

Based on the pK_a values predicted by the PROPKA web server [6], several charged residues of Pgp (K822, E913, and E1080) were initially modeled as neutral during the equilibrating phase. However, all three residues became solvent-exposed after 4.5 ns of simulations, and thus interpreted as charged upon repeated pK_a prediction using the PROPKA server. As a result, all Pgp residues were modeled in their default protonation states during the production simulations.

To generate membrane-embedded Pgp models, the Pgp structure was first reoriented to align its long axis with the z-axis of the simulation system, and the axis connecting two NBDs with the y-axis. The structure was then placed into a POPE bilayer of 100×130 Å^2 dimension, where lipid molecules in close contact with protein structure (any atom, including hydrogens, within 0.6 Å cutoff) removed from the system. The resulting bilayer is composed of ∼300 lipid molecules (typically ∼155 in the extracellular leaflet and ∼145 in the cytoplasmic one). The conformation and dynamics of a membrane-embedded protein in MD simulations can be affected by its initial lipid contacts, which in turn, depend on how the protein was initially inserted into the lipid bilayer. In order to enhance the conformational sampling of the membrane-embedded Pgp, and ensure that our conclusions regarding its dynamics are not affected by the initial membrane placement, four independent simulation systems of membrane-embedded Pgp were generated (Systems 1 to 4). Using System 1 as a structural reference, the Pgp structure was rotated 180 °, 30 °, and 330 ° about the bilayer normal axis prior to its membrane insertion in Systems 2, 3 and 4, respectively. In addition to the membrane-embedded transporter, each of the simulation systems also contained ∼48,750 water molecules, ∼90 K^+ ions, and ∼90 Cl^- ions. Overall, each simulation system contained ∼200,000 atoms with dimensions of $100 \times 130 \times 170$ Å^3 prior to equilibration.

Simulation Protocols

Unless described otherwise, all the MD simulations were performed using NAMD 2.6 [7], with the force field CHARMM27 used for protein [8], lipid [9], and nucleic acids [10]. The TIP3P model was adopted for water molecules [11]. The simulations were performed at 310 K, and the constant temperature was maintained with Langevin dynamics using a damping coefficient of $\gamma = 0.5\,\mathrm{ps}^{-1}$. Constant pressure (1.01325 bar) was maintained only along the z-axis (bilayer normal) of the simulation system using the Nosé-Hoover Langevin piston method [12, 13], while the system dimensions in the xy-plane were fixed ($\mathrm{NP}_n\mathrm{AT}$ ensemble, except for initial equilibration as described below). A 10 Å switching distance and a 12 Å cutoff distance were used for the calculation of non-bonded interactions, and the particle mesh Ewald (PME) method [14] was employed to calculate the long-range electrostatic interactions. All the simulations used a 2 fs integration time unless specified otherwise.

The equilibration of the membrane-embedded Pgp started with "melting" of the lipid tails, *i.e.*, constraining all atoms except the acyl chains of the lipid molecules. After 0.5 ns melting/mixing of the lipid tails, the four systems were further simulated under constant pressure conditions (NPT ensembles), during which only the protein atoms were harmonically restrained (5 kcal/mol·Å^2). The protein-restrained simulation was performed for at least 2.5 ns for each system, in order to allow the lipid bilayer to adjust its area and optimally pack against the TMDs of Pgp. The constant-pressure simulation was continued for additional 1–2 ns after the removal of all the restraints, until the membrane area converged into a relatively stable size without transforming into a gel phase (monitored by calculating the order parameter S_{CD} of the acyl chains, data not shown). All the equilibration simulations were performed using 1 fs integration time steps. After the initial equilibration, 50 ns of production simulation were performed (using 2 fs time steps) for each of the four systems using the general protocols described above, and the trajectories were recorded every 5 ps.

Mg-ATP Docking at the Walker A Motifs

During the transport cycle of Pgp, major structural transitions is accompanied/induced by the binding of Mg-ATP. Therefore, in addition to the *apo* state, it is desirable to also study a nucleotide-bound form of Pgp. A model for this form can be generated by the docking of Mg-ATP to the nucleotide binding sites. However, the conformations of the Walker A motifs in ABC transporters differ drastically between the Mg-ATP-bound and the nucleotide-free states (see Fig. 1, notice the overlapping structures between the protein and the Mg-ATP to be docked). In order to accurately model the nucleotide-bound state and to prevent rapid nucleotide dissociation during the production simulations, which can easily arise upon incorrectly modeling the nucleotide in its binding site, one must carefully modify the conformation near the Walker A motif during the Mg-ATP docking processes.

Taking advantage of the exceptionally high sequence identity of the Walker A motif among all ABC transporters, and the remarkably similar interactions between the bound nucleotide and the conserved binding site residues, the crystal structure of the dimeric, Mg-ATP-bound NBD of the H662A mutant of the *Escherichia coli* hemolysin exporter, HlyB

(PDB: 1XEF [15]), was used as a structural template to generate Mg-ATP-bound nucleotide binding sites in Pgp. To obtain the position of the docked Mg-ATP relative to the nucleotide binding residues of Pgp, the two NBDs of Pgp were individually superimposed with the chain A of the HlyB-NBD structure, and the coordinates of the Mg-ATP were copied from the HlyB structure into the Pgp model. Specifically, the C_α atoms at the two secondary structure elements flanking the Walker A motif were used for the superposition (Q417–G423 and G428–R438 of Pgp-NBD1; Q1060–G1066 and G1071–R1081 of Pgp-NBD2; and E496–G502 and G507–R517 of HlyB). The alignment and Mg-ATP docking were done for Systems 1 to 4 individually, using their structures obtained after the initial equilibration phase (details below).

To transform the conformation of the Walker A motif into a nucleotide-bound arrangement, distance restraints were employed between certain atoms of Mg-ATP and their binding residues in Pgp. These inter-atomic distance restraints were introduced to the systems in the form of extra chemical bonds, a feature provided in a then beta version of NAMD 2.7 (more at http://www.ks.uiuc.edu/Research/namd/2.7/features.html). These "extrabonds" were used only for 1.5 ns to adjust the conformation of the nucleotide binding sites (details below) and were not employed in the following production simulations.

Except for the ring-stacking interactions between the adenine ring of ATP and the aromatic side chain of Y397/Y1040 of Pgp (arbitrarily set at 3.5 Å), the equilibrium distances used for the extrabonds were derived from the corresponding distances in the HlyB-NBD crystal structure [15] (averaged from four NBD monomers in the asymmetric unit). The spring constant of the extrabonds were set according to the observed distances in the HlyB-NBD structure: a bond shorter than 2.9 Å was restrained with a higher spring constant $k = 61.6$ kcal/mol·Å^2 ($\sim$100 $k_B T$/Å^2 at 310 K); a bond between 2.9–3.1 Å was given a moderate spring constant $k = 30.8$ kcal/mol·Å^2 ($\sim$50 $k_B T$/Å^2); and a bond longer than 3.1 Å was given a $k = 12.3$ kcal/mol·Å^2 ($\sim$20 $k_B T$/Å^2). The Mg^{2+} coordinating interactions (typically shorter than 2.2 Å), on the other hand, were constrained with a force constant of $k = 250$ kcal/mol·Å^2, analogous to the Fe^{2+} coordinating interactions defined for heme in the CHARMM force field [16]. All the extrabond parameters for Mg-ATP docking are listed in Table S1 and their positions are graphically represented in Fig. S1a.

The simulations used for Mg-ATP docking were performed with 1 fs time steps. The simulations started with 3000 steps of energy minimization followed by three stages of MD, each 0.5 ns. During the first stage, positional restraints of $k = 5$ kcal/mol·Å^2 were applied to all heavy atoms of lipid molecules and all C_α atoms in the TMDs of Pgp (V33–K368 and W694–K1010), while the coordinates of the docked Mg-ATP were fixed, so that only the NBD conformations could change. In the second stage, all the fixed-atom constraints and the harmonic positional restraints applied to the TMD/lipids were removed, but the extrabonds between Mg-ATP and the binding sites were retained. In the last stage, the spring constants of the extrabonds were reduced by five folds to allow the nucleotide binding sites to be more flexible. The extrabonds were completely removed at $t = 1.5$ ns and equilibrium simulations were continued until $t = 7.5$ ns. Simulations beyond $t = 7.5$ ns (until $t = 50$ ns) were integrated using 2 fs time steps.

Data Analysis

The program VMD [17] was used for analysis and visualization of the trajectories. The inter-NBD distance is defined as the distance between the centers of masses (CoMs) of the two NBDs (NBD1: N387–T626; NBD2: N1030–A1271, hydrogen atoms included). The distance between the nucleotide binding motifs at the two nucleotide binding sites is defined using the hydrogen-excluded CoMs of the following two sets: a) the Walker A motif of NBD1 (G423–S430) and the LSGGQ motif of NBD2 (L1172–R1179); b) the LSGGQ motif of NBD1 (L527–R534) and the Walker A motif of NBD2 (G1066–S1073). The integrity of transmembrane helices during the simulations was examined by monitoring the hydrogen bonds between residues i and $i+4$, using a distance criterion of less 4.0 Å between the donor (nitrogen) and acceptor (oxygen) atoms, and an N–H–O angle greater than 150°. The theoretical temperature factors for each C_α atoms are derived from their observed RMSF in the simulations, using the formula $\theta = (8\pi^2/3)\times \mathrm{RMSF}^2$.

Structure-based sequence alignment was performed using the Multiseq plugin [18] of VMD. Crystal structures of inward-facing Pgp (PDB:3G5U [1]), outward-facing SAV1866 (PDB:2HYD [19]), outward-facing MsbA of *Salmonella typhimurium* (PDB:3B60 [20]), inward-facing TM287/288 (PDB:3QF4 [21]), and inward-facing ABCB10 (PDB:4AYT, Pike *et. al*, to be published) were used for the structure based alignment. Although MsbA proteins from two other species have been crystallized in different conformations (PDB:3B5W and PDB:3B5X [20]), due to their lower resolutions these structures were not included in the alignment. To structurally align all the ABC exporters in different conformational states, their sequences were initially aligned using the ClustalW method [22]. Based on the secondary structures of the aligned sequences, the TMD structures of those ABC exporters were divided into several sections (the "elbow helix" with TM1/7 and TM2/8; TM3/9 alone; TM4/10 with TM5/11; TM6/12 alone) and each section was individually structurally aligned using the STAMP method [23]. The multiple alignment was then formatted using the Sequence Manipulation Suite [24] web server.

The Gly/Pro contents in the TMDs of ABC exporters were analyzed over 33,741 aligned sequences retrieved from the database Pfam [25] (family "ABC_membrane", entry PF00664). Only residues in the globally aligned regions are counted, so that the distribution of Gly/Pro content not be biased by proteins containing extra domains, additional transmembrane helices, and/or lengthy loops between transmembrane helices. Note that based on the sequences with resolved structures in the Pfam alignment, it is found that the aligned region in this database entry does not cover the elbow helix and the cytoplasmic part of helix TM6/12, so that the Gly/Pro contents might be systematically underestimated. However, the Gly/Pro content comparison among different ABC exporters should not be affected significantly by the incomplete coverage of the alignment.

References

[1] Aller S.G., Yu J., Ward A., Weng Y., Chittaboina S., Zhuo R., Harrell P.M., Trinh Y.T., Zhang Q., Urbatsch I.L., and Chang G. (2009) Structure of P-glycoprotein reveals a molecular basis for poly-specific drug binding *Science* **323**, 1718–1722

[2] Loo T.W., and Clarke D.M. (1994) Reconstitution of drug-stimulated ATPase activity following co-expression of each half of human P-glycoprotein as separate polypeptides *J. Biol. Chem.* **269**, 7750–7755

[3] Nuti S.L., Mehdi A., and Rao U.S. (2000) Activation of the human P-glycoprotein ATPase by trypsin *Biochemistry* **39**, 3424–3432

[4] Nuti S.L., and Rao U.S. (2002) Proteolytic cleavage of the linker region of the human P-glycoprotein modulates its ATPase function *J. Biol. Chem.* **277**, 29417–29423

[5] Sato T., Kodan A., Kimura Y., Ueda K., Nakatsu T., and Kato H. (2009) Functional role of the linker region in purified human P-glycoprotein *FEBS J.* **276**, 3504–3516

[6] Li H., Robertson A.D., and Jensen J.H. (2005) Very fast empirical prediction and interpretation of protein pKa values *Proteins: Struct., Func., Bioinf.* **61**, 704–721

[7] Phillips J.C., Braun R., Wang W., Gumbart J., Tajkhorshid E., Villa E., Chipot C., Skeel R.D., Kale L., and Schulten K. (2005) Scalable molecular dynamics with NAMD *J. Comp. Chem.* **26**, 1781–1802

[8] MacKerell, Jr. A.D., Feig M., and Brooks, III C.L. (2004) Extending the treatment of backbone energetics in protein force fields: Limitations of gas-phase quantum mechanics in reproducing protein conformational distributions in molecular dynamics simulations *J. Comp. Chem.* **25**, 1400–1415

[9] Feller S.E., and MacKerell, Jr. A.D. (2000) An improved empirical potential energy function for molecular simulations of phospholipids *J. Phys. Chem. B* **104**, 7510–7515

[10] Foloppe N., and MacKerrell Jr. A.D. (2000) All-atom empirical force field for nucleic acids: I. parameter optimization based on small molecule and condensed phase macromolecular target data *J. Comp. Chem.* **21**, 86–104

[11] Jorgensen W.L., Chandrasekhar J., Madura J.D., Impey R.W., and Klein M.L. (1983) Comparison of simple potential functions for simulating liquid water *J. Chem. Phys.* **79**, 926–935

[12] Martyna G.J., Tobias D.J., and Klein M.L. (1994) Constant pressure molecular dynamics algorithms *J. Chem. Phys.* **101**, 4177–4189

[13] Feller S.E., Zhang Y., Pastor R.W., and Brooks B.R. (1995) Constant pressure molecular dynamics simulation: The Langevin piston method *J. Chem. Phys.* **103**, 4613–4621

[14] Darden T., York D., and Pedersen L.G. (1993) Particle mesh Ewald: An $N \cdot \log(N)$ method for Ewald sums in large systems *J. Chem. Phys.* **98**, 10089–10092

[15] Zaitseva J., Jenewein S., Jumpertz T., Holland I.B., and Schmitt L. (2005) H662 is the linchpin of ATP hydrolysis in the nucleotide-binding domain of the ABC transporter HlyB *EMBO J.* **24**, 1901–1910

[16] MacKerell A.D. Jr., Bashford D., Bellott M., Dunbrack J.R.L., Evanseck J., Field M.J., Fischer S., Gao J., Guo H., Ha S., Joseph D., Kuchnir L., Kuczera K., Lau F.T.K., Mattos C., Michnick S., Ngo T., Nguyen D.T., Prodhom B., Roux B., Schlenkrich M., Smith J., Stote R., Straub J., Watanabe M., Wiorkiewicz-Kuczera J., Yin D., and Karplus M. (1992) Self-consistent parameterization of biomolecules for molecular modeling and condensed phase simulations *FASEB J.* **6**, A143–A143

[17] Humphrey W., Dalke A., and Schulten K. (1996) VMD – Visual Molecular Dynamics *J. Mol. Graphics* **14**, 33–38

[18] Roberts E., Eargle J., Wright D., and Luthey-Schulten Z. (2006) MultiSeq: Unifying sequence and structure data for evolutionary analysis *BMC Bioinformatics* **7**, 382

[19] Dawson R.J., and Locher K.P. (2006) Structure of a bacterial multidrug ABC transporter *Nature* **443**, 180–185

[20] Ward A., Reyes C.L., Yu J., Roth C.B., and Chang G. (2007) Flexibility in the ABC transporter MsbA: Alternating access with a twist *Proc. Natl. Acad. Sci. USA* **104**, 19005–19010

[21] Hohl M., Briand C., Grütter M.G., and Seeger M.A. (2012) Crystal structure of a heterodimeric ABC transporter in its inward-facing conformation *Nat. Struct. Mol. Biol.* **19**, 395–402

[22] Larkin M.A., Blackshields G., Brown N.P., Chenna R., McWilliam P.A.M.H., Valentin F., Wallace I.M., Wilm A., Lopez R., Thompson J.D., Gibson T.J., and Higgins D.G. (2007) Clustal W and Clustal X version 2.0 *Bioinformatics* **23**, 2947–2948

[23] Russell R.B., and Barton G.J. (1992) Multiple protein sequence alignment from tertiary structure comparison: assignment of global and residue confidence levels. *Proteins: Struct., Func., Gen.* **14**, 309–323

[24] Stothard P. (2000) The sequence manipulation suite: JavaScript programs for analyzing and formatting protein and DNA sequences *Biotechniques* **28**, 1102–1104

[25] Punta M., Coggill P.C., Eberhardt R.Y., Mistry J., Tate J., Boursnell C., Pang N., Forslund K., Ceric G., Clements J., Heger A., Holm L., Sonnhammer E.L.L., Eddy S.R., Bateman A., and Finn R.D. (2012) The Pfam protein families database *Nucleic Acids Res.* **40**, D290–D301

Atom-1		Force Constant	Equilibrium	Atom-2	
Residue	Atom	(kcal·mol^{-1}·Å^{-2})	Distance (Å)	Residue	Atom
Y397/1040	CG	12.320	3.50	ATP	C2
Y397/1040	CD1	12.320	3.50	ATP	N3
Y397/1040	CD2	12.320	3.50	ATP	N1
Y397/1040	CE1	12.320	3.50	ATP	C4
Y397/1040	CE2	12.320	3.50	ATP	C6
Y397/1040	CZ	12.320	3.50	ATP	C5
Y397/1040	OH	12.320	3.50	ATP	N7
Y397/1040	OH	61.600	2.74	T431/1074	OG1
N424/S1067	O	30.800	2.95	K429/1072	NZ
S425/1068	OG	61.600	2.71	ATP	O1G
G426/1069	N	61.600	2.76	ATP	O3B
G428/1071	N	12.320	3.10	ATP	O1B
K429/1072	NZ	61.600	2.81	ATP	O2G
K429/1072	NZ	61.600	2.88	ATP	O1B
S430/1073	N	30.800	3.04	ATP	O2B
S430/1073	OG	250.000	2.22	Mg^{2+}	Mg^{2+}
T431/1074	OG1	61.600	2.67	ATP	O2A
D551/1196	OD1	61.600	2.84	H_2O–(1)	O
ATP	O2B	250.000	2.11	Mg^{2+}	Mg^{2+}
ATP	O3G	250.000	1.87	Mg^{2+}	Mg^{2+}
Mg^{2+}	Mg^{2+}	250.000	2.17	H_2O–(1)	O
Mg^{2+}	Mg^{2+}	250.000	2.04	H_2O–(2)	O
Mg^{2+}	Mg^{2+}	250.000	2.04	H_2O–(3)	O

Table S1: The extrabond parameters applied during the first 0.5 ns of Mg-ATP docking. The positions of the three Mg^{2+}-bound water molecules (numbered 1–3) are labeled in Fig. S1a.

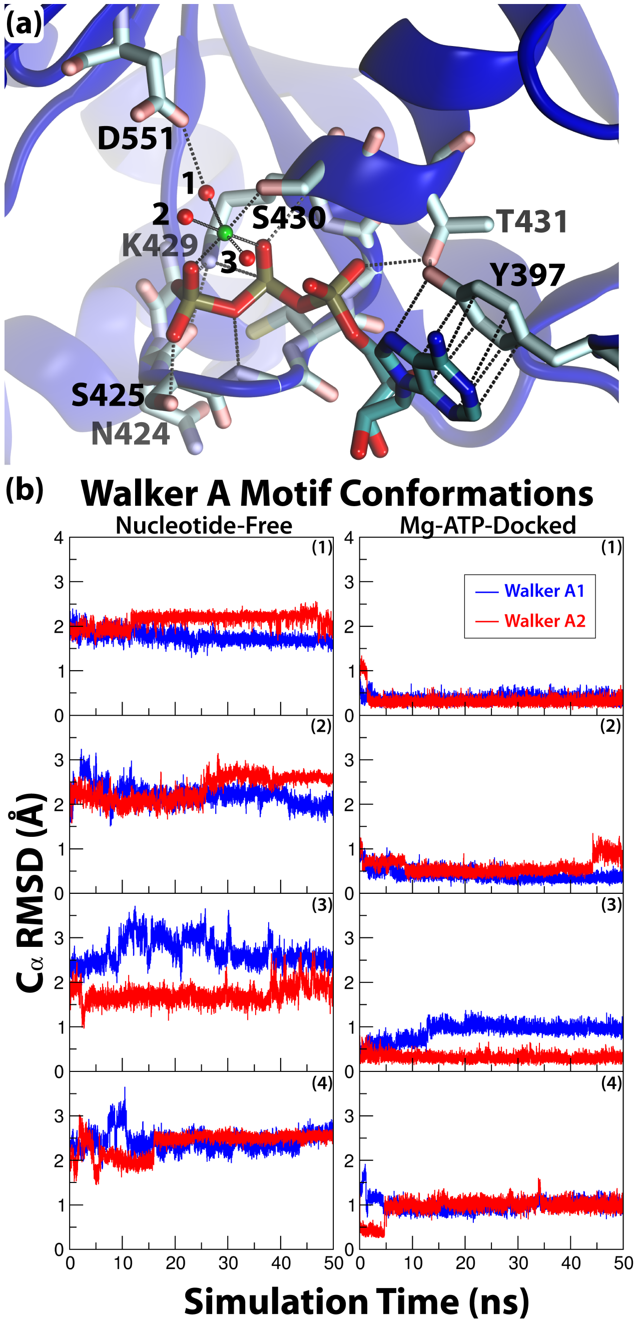

Figure S1: The docking of Mg-ATP at the Walker A motif. (a) Close-up view of a Mg-ATP-docked nucleotide binding site (NBD1 at t = 0.5 ns of System 1). The configurations are similar in NBD2 and in all other simulation systems. The stick models are used for ATP and residues of the Walker A motif, the A-loop tyrosine and the Walker B aspartate. Mg^{2+} is drawn as a green sphere and its coordinating water in red. The "extrabonds" terms listed for Mg-ATP-docking simulations are shown as dotted lines. (b) Structural dynamics of the Walker A motif during the simulations. The Walker A motifs of Pgp (G423–T431 and G1066–T1074) were superimposed individually on the reference structure (HlyB:G502–T510 from chain A of 1XEF [15]), and the C_α RMSD was recorded over all trajectories.

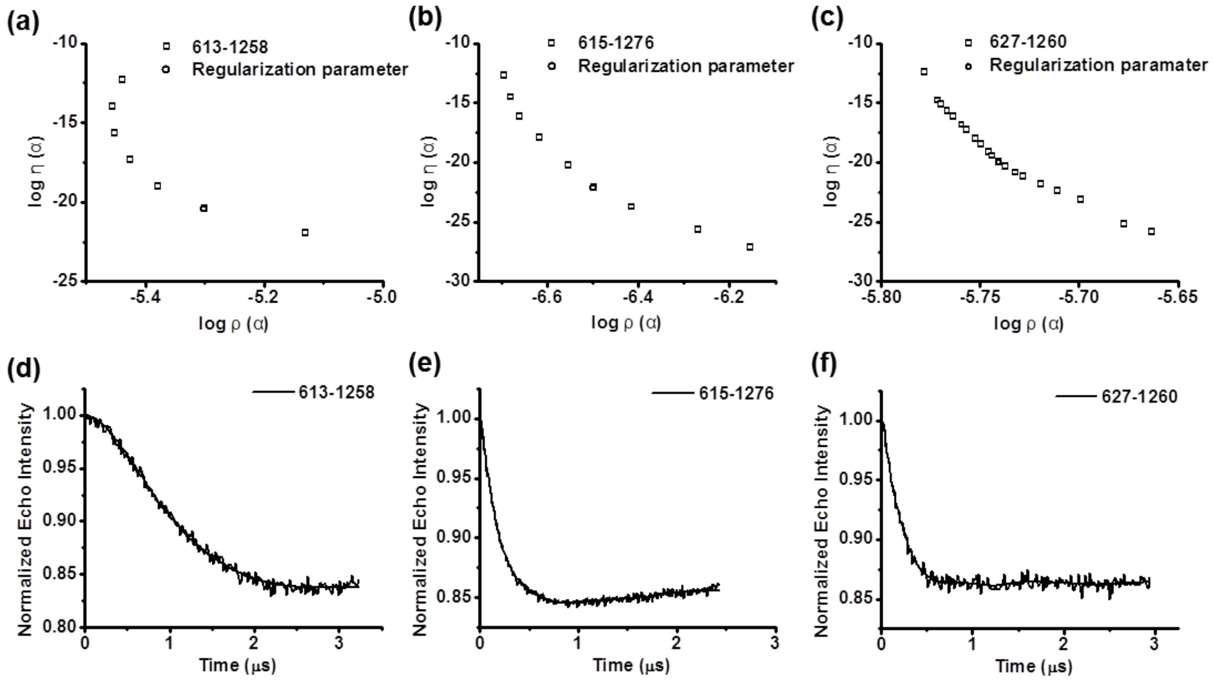

Figure S2: (a–c) The regularization parameter chosen from the L-curve from Tikhonov analysis is indicated as a circle for the distance distributions in Fig. 2c. (d–f) Time-dependent decays of DEER signal and fits determined by Tikhonov regularization shown in (a–c).

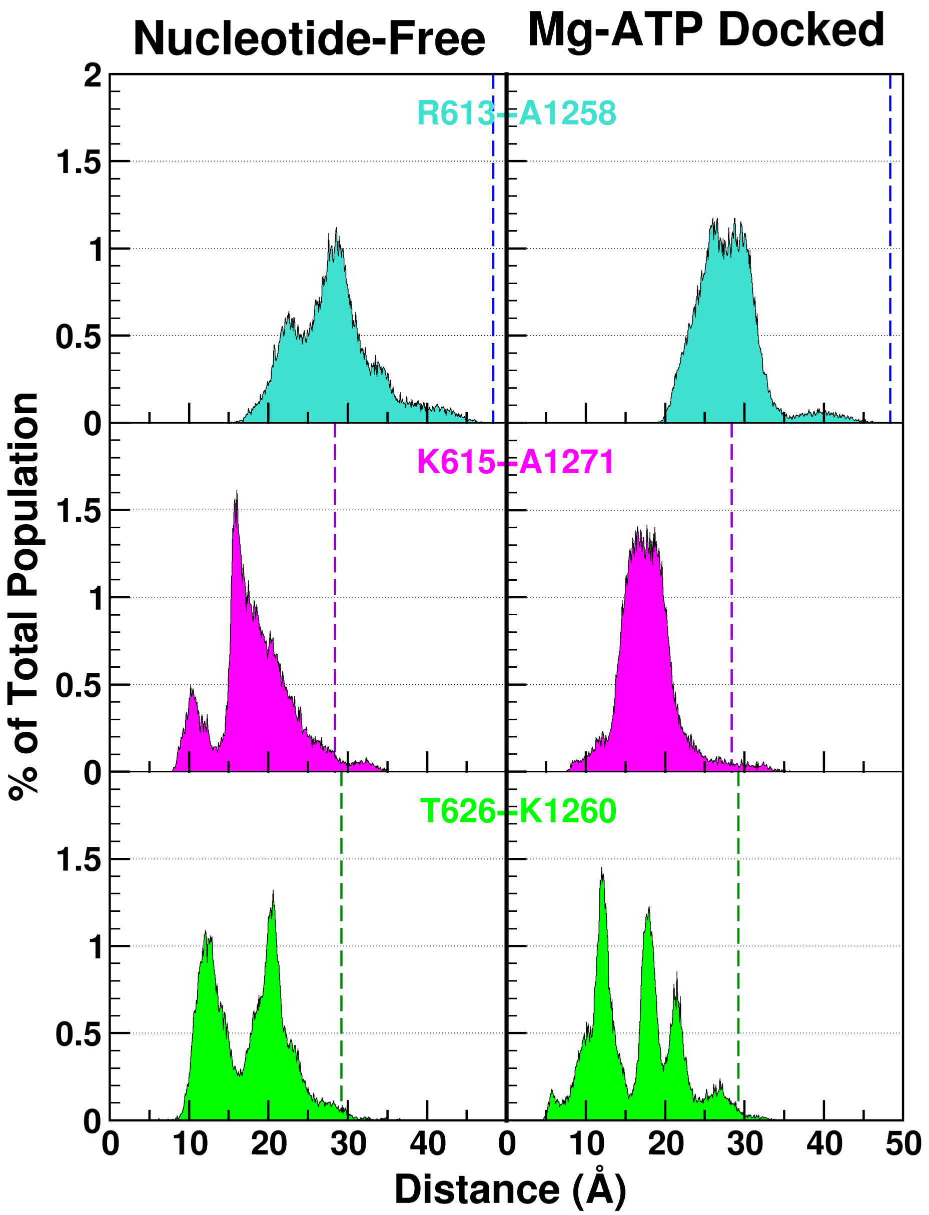

Figure S3: Distributions of the C_α-C_α distances between the spin-labeled residues (R613–A1258) or the closest residues resolved in the crystal structure (K615–A1271 and T626–K1260) captured in the *apo* (left) or Mg-ATP-docked (right) simulations. The distances observed in the crystal structure are marked in dashed lines, and the positions of the spin-labeled residues are shown in Fig. 1.

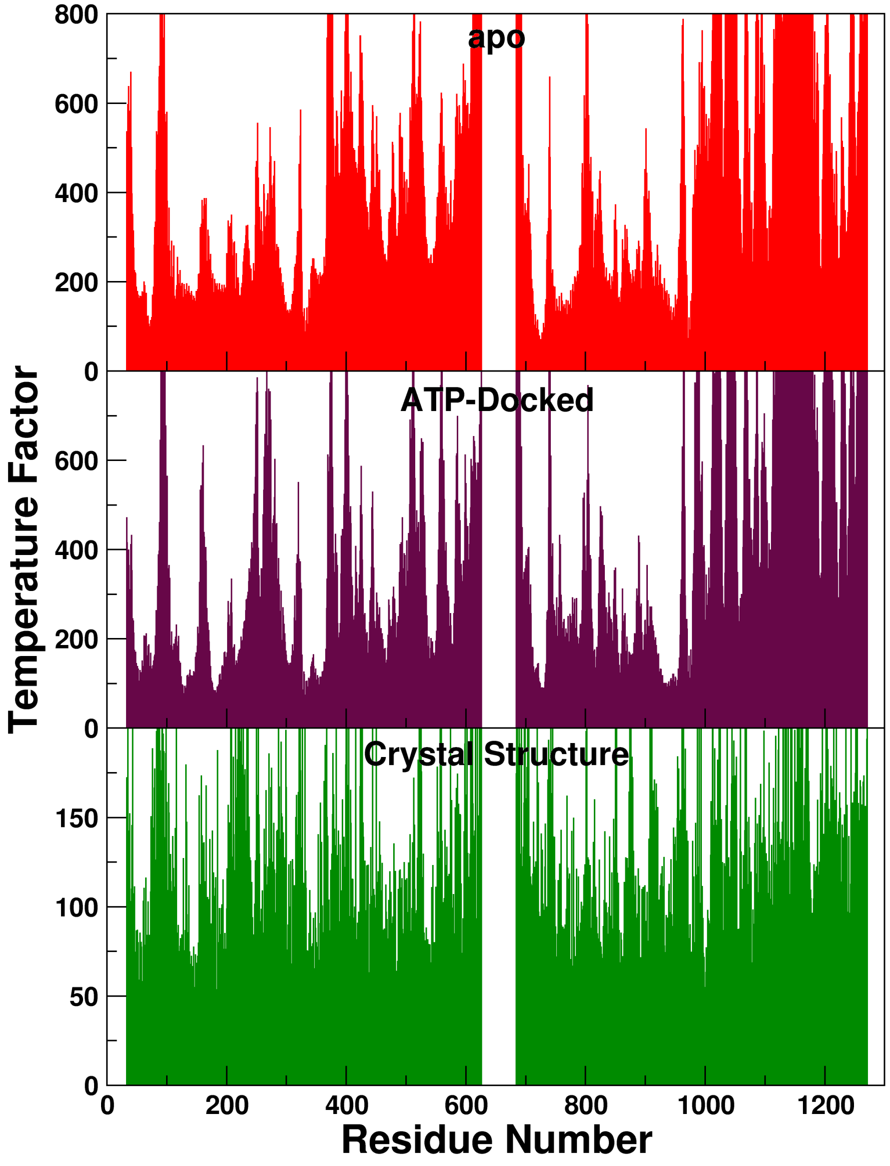

Figure S4: The temperature factor of C_α atoms derived from their RMSF in the simulations using the formula $\theta = (8\pi^2/3)\times \mathrm{RMSF}^2$. The crystallographic temperature factors are also included for reference, note the y-scale is different in the bottom panel.

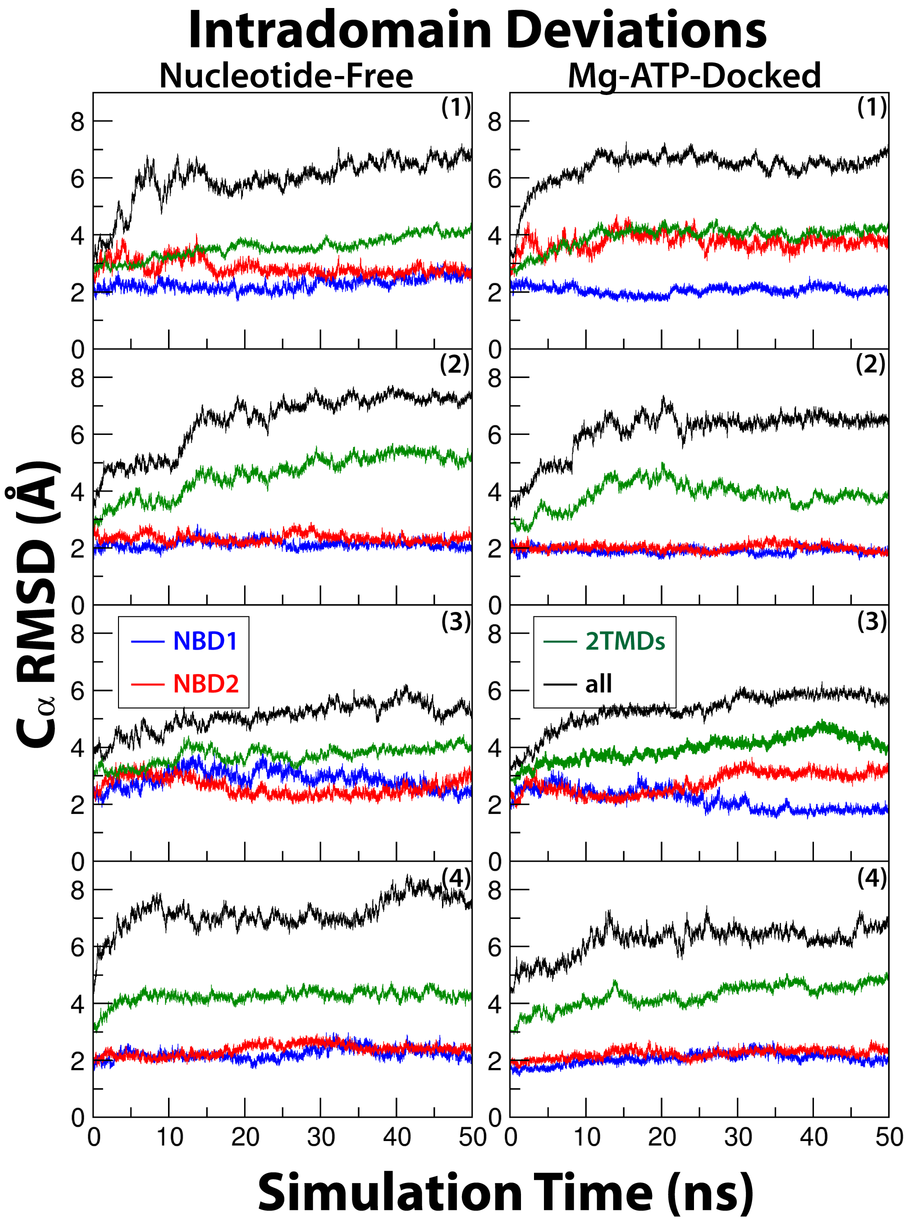

Figure S5: RMSD of the entire Pgp structure (black) and of individual domains of Pgp (blue: NBD1, red: NBD2, green: combined two TMDs) during the simulations under nucleotide-free or Mg-ATP-docked conditions.

```
Pgp-N     VSVLTMFRYAG-WLDRLYMLVGTLAAIIHGVALPLMMLIFGDMTDS-F-A-SVGN-----  83
Pgp-C     WRILKLNSTEW-P--Y--FVVGIFCAIINGGLQPAFSVIFSKVVGV-F-TNGG-------  739
ABCB10    LPEARKLLGLAYP-ERRRLAAAVGFLTMSSVISMSAPFFLGKIIDV-I-Y-TNP------  203
TM287     -SK--TLARYLKP-YWIFAVLAPLFMVVEVICDLSQPTLLARIVDEGI-A-R--------  46
TM288     TATLRRLLGYLRP-HTFTLIMVFVFVTVSSILGVLSPYLIGKTIDVVF-V-P--------  70
SAV1866   --MIKRYLQFVKP-----YKYRIFATIIVGIIKFGIPMLIPLLIKY-A-IDGVIN-NHAL  50
MsbA      WQTFRRLWPTIAP-----FKAGLIVAGIALILNAASDTFMLSLLKP-LLD-DGFGK----  58
CONS_SS   --HHHHH-----------HHHHHHHHHHHHHHHHHHHHHHHHHHHH--------------

Pgp-N     V--SKNSTNMSEADKRAMFAKLEEE-MTTYAYYYTGIGAGVLIVAYIQVSFWCLAAGRQI  140
Pgp-C     PPE-----------------TQ-RQNSNLFSLLFLILGIISFITFFLQGFTFGKAGEILT  781
ABCB10    TVD-------------YSDNLT-RLCLGLSAVFL-CGAAANAIRVYLMQTSGQRIVNRLR  248
TM287     GD---------------FSLVL-KTGILMLIVAL-IGAVGGIGCTVFASYASQNFGADLR  89
TM288     RR---------------FDLLP-RYMLILGTIYA-LTSLLFWLQGKIMLTLSQDVVFRLR  113
SAV1866   T------------TDEKVHHLT-IAIGIALFIFVIVRPPIEFIRQYLAQWTSNKILYDIR  97
MsbA      TD---------------------RSVLLWMPLVVIGLMILRGITSYISSYCISWVSGKVV  97
CONS_SS   -----------------------HHHHHHHHHHH-HHHHHHHHHHHHHHHHHHHHHHHHH

Pgp-N     HKIRQKFFHAIMNQE-IGWFDVHDV--GELNTRLTDDVSKINEGIGDKI-GM-FF-QAMA  194
Pgp-C     KRLRYMVFKSMLRQD-VSWFDDPKNTTGALTTRLANDAAQVKGATGSRL-AV-IF-QNIA  837
ABCB10    TSLFSSILRQ--E-V-AFFD-KTR------TGELINRLSSDTALLGRSVTEN-LS-DGLR  295
TM287     RDLFRKVLSF--S-I-SNVN-RFH------TSSLITRLTNDVTQLQNLVM-M-LLRIVVR  136
TM288     KELFEKLQRV--P-V-GFFD-RTP------HGDIISRVINDVDNINNVLGNS-II-QFFS  160
SAV1866   KKLYNHLQAL--S-A-RFYA-NNQ------VGQVISRVINDVEQTKDFI-LTGLM-NIWL  144
MsbA      MTMRRRLFGHMMGMPVAFFD-KQS--TGTLLSRITYDSEQVASSSSGAL-ITVVR-EGAS  152
CONS_SS   HHHHHHHHH---------------------HHHHHHHHHHHHHHHHHHH-HH-HH-HHHH

Pgp-N     TFFGG-FIIG-FTR---GWK----LTLVILAISPVLG-LSAGIWAKILSSFTDKELHAYA  244
Pgp-C     NLGTG-IIIS-LIY---G-WQL-------TLLLLAIV-PIIAIAGVVEMKMLSGQALKDK  883
ABCB10    AGAQA-SVGI-SMMFFVS--------PNLATFVLSVVPPVSIIAV-IYGRYLRKLTKVTQ  344
TM287     APLLF-VGGI-VMAVSIN--------VKLSSVLIFLI-PPIVLLFVWLTKKGNPLFRKIQ  185
TM288     GIVTL-AGAV-IMMFRVN--------VILSLVTLSIV-PLTVLITQIVSSQTRKYFYENQ  209
SAV1866   DCITI-IIAL-SIMFF-L-D--VK----LTLAALFIF-PFYILTVYVFFGRLRKLTRERS  193
MsbA      IIG-LFIM-MFYY------S--WQ----LSIILVVLA-PIVSIAIRVVSKRFRSISKNMQ  197
CONS_SS   HHHHH-HH-H-HH---------------HHHHHHHHH-HHHHHHHHHHHHHHHHHHHHHH

Pgp-N     KAGAVAE-E--V-LAAIRTVIAF-GGQ----KKELERYNNNLEEAKRLGIKKAITANISM  295
Pgp-C     KELEGSGKIATEAIENF-RTVVSLT-REQKFETMYAQSLQ-IPYRNAMKK---AHVFGIT  937
ABCB10    DSLAQATQLAEERIGNVRTVRAF-G-KEMTEIEKYASKVD-HVMQLARKE---AFARAGF  398
TM287     ESTDEVNRVVRENLLGVRVVRAF-R-REEYENENFRKANE-SLRRSIISA---FSLIVFA  239
TM288     RVLGQLNGIIEEDISGLTVIKLF-T-REEKEMEKFDRVNE-SLRKVGTKA---QIFSGVL  263
SAV1866   QALAEVQGFLHERVQGISVVKSF-A-IEDNEAKNFDKKNT-NFLTRALKH---TRWNAYS  247
MsbA      NTMGQVTTSAEQMLKGHKEVLIF-G-GQEVETKRFDKVSN-KMRLQGMKM---VSASSIS  251
CONS_SS   HHHHHHHHHHHHHHH-HHHHHH------HHHHHHHHHHHH-HHHHHHHHH---HHHHHHH

Pgp-N     GAAFLLIYASYALAFWYGTSLVISK--EYSIG-Q-VLTVFFSVLIGAFSVGQASP-NIEA  350
Pgp-C     FSFTQAMMYFSYAAAFRFGAYLVTQQLMTFE---NVLL-VFSAIVFGAMAVGQVSSFAPD  993
ABCB10    FGATGLSGNLIVLSVLYKGGLLMGSA-HMT------VGELSSFLMYAFWVGISIG-GLSS  450
TM287     LPLFIFIVNMGMIAVLWFGGVLVRNN-QME------IGSIMAYTNYLMQIMFSLM-MIGN  291
TM288     PPLMNMVNNLGFALISGFGGWLALKD-IIT---VGTIATFIGYSRQFTRPLNELSNQFNM  319
SAV1866   FAAINTVTDIGPIIVIGVGAYLAISG-SIT--VGTLAAFVGYLELLFGPLRRLVA-SFTT  303
MsbA      DPIIQLIASLALAFVLYAASFPSVMD-SLTA--GTITVVFSSMIALMRPLKSLTN-VNAQ  307
CONS_SS   HHHHHHHHHHHHHHHHHHHHH---------------HHHHHHHHHHHHHHHHHHH-HHHH

Pgp-N     FANARGAAYEVFKIIDN  367
Pgp-C     YAKATVSASHIIRIIEK  1010
ABCB10    FYSELMKGLGAGGRLWE  467
TM287     ILNFIVRASASAKRVLE  308
TM288     IQMAL-ASAERIFEILD  335
SAV1866   LTQSFASMDRVFQLIDE  320
MsbA      FQRGMAACQTLFAILDS  324
CONS_SS   HHHHHHHHHHHHHHHH-
```

Figure S6: Structure based sequence alignment of TMDs of known ABC exporter structures. The position of glycine and proline residues near the helical unwinding regions shown in Fig. 4a are highlighted in red, and the conserved secondary structure elements (namely, the "elbow" helix and transmembrane helices TM1/7 to TM6/12) were labeled in yellow.

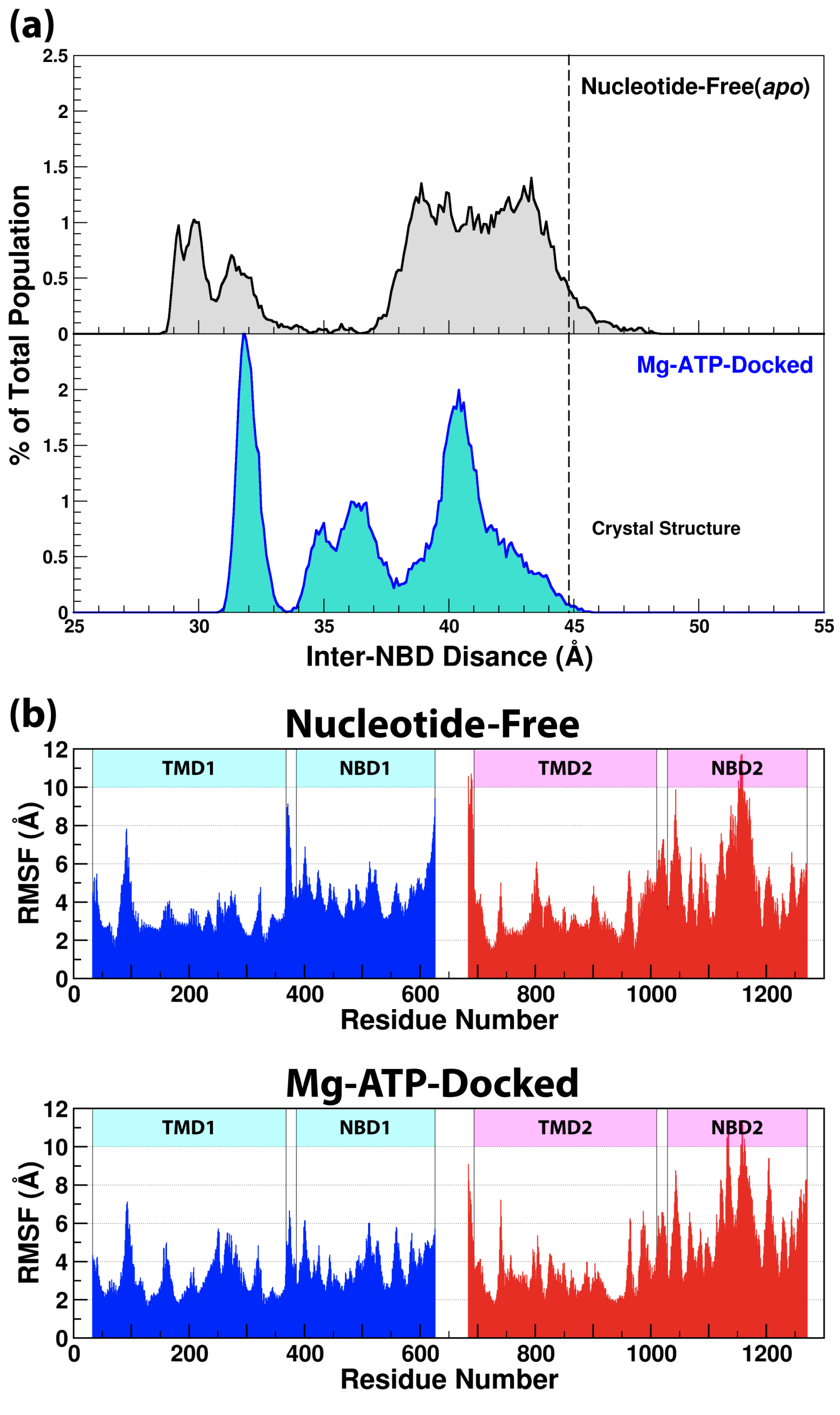

Figure S7: Analysis of Pgp dynamics after excluding the first 10 ns of the production trajectories. Neither the distributions of inter-NBD distances (a), nor the RMSF of C_α atoms (b) are significantly changed when the trajectories between $t = 0$ and $t = 10$ ns were excluded from the calculations.
